# Simultaneous in-field boost for patients with 1 to 4 brain metastasis/es treated with volumetric modulated arc therapy: a prospective study on quality-of-life

**DOI:** 10.1186/1748-717X-6-79

**Published:** 2011-06-30

**Authors:** Damien C Weber, Francesca Caparrotti, Mohamed Laouiti, Karim Malek

**Affiliations:** 1 Radiation Oncology Department, Département de l'Imagerie Médical et Science de l'Information (DIMSI), Geneva University Hospital/University of Geneva, CH-1211 Geneva 14, Switzerland; 2 University of Geneva, CH-1211 Geneva, Switzerland

## Abstract

**Purpose:**

To assess treatment toxicity and patients' survival/quality of life (QoL) after volumetric modulated arc therapy (VMAT) with simultaneous in-field boost (SIB) for cancer patients with 1 - 4 brain metastases (BM) treated with or without surgery.

**Methods and Materials:**

Between March and December 2010, 29 BM patients (total volume BM, < 40 cm^3^) aged < 80 years, KPS ≥ 70, RPA < III were included in this prospective trial. Whole brain VMAT (30 Gy) and a SIB to the BM (40 Gy) was delivered in 10 fraction. Mean age was 62.1 ± 8.5 years. Fifteen (51.7%) underwent surgery. KPS and MMSE were prospectively assessed. A self-assessed questionnaire was used to assess the QoL (EORTC QLQ-C30 with -BN20 module).

**Results:**

As of April 2011 and after a mean FU of 5.4 ± 2.8 months, 14 (48.3%) patients died. The 6-month overall survival was 55.1%. Alopecia was only observed in 9 (31%) patients. In 3-month survivors, KPS was significantly (*p *= 0.01) decreased. MMSE score remained however stable (*p *= 0.33). Overall, QoL did decrease after VMAT. The mean QLQ-C30 global health status (*p *= 0.72) and emotional functional (*p *= 0.91) scores were decreased (low QoL). Physical (*p *= 0.05) and role functioning score (*p *= 0.01) were significantly worse and rapidly decreased during treatment. The majority of BN20 domains and single items worsened 3 months after VMAT except headaches (*p *= 0.046) and bladder control (*p *= 0.26) which improved.

**Conclusions:**

The delivery of 40 Gy in 10 fractions to 1 - 4 BM using VMAT was achieved with no significant toxicity. QoL, performance status, but not MMSE, was however compromised 3 months after treatment in this selected cohort of BM patients.

## Introduction

Brain metastases (BMs) occur in 25 to 45% of all cancer patients and represent thus a significant clinical problem in cancer management [1]. Whole brain radiation therapy (WBRT) with steroids is usually the primary treatment option for patients with multiple BMs. Patients with 1 - 4 BMs are routinely treated with surgery and/or radiosurgery, with or without WBRT. After these treatments, local and distant brain failure is however a clinical issue and occurs in a substantial number of patients. Two prospective phase III trial have shown a 1-year local and/or brain failure rate of 30% - 100% [2,3].

Improvement of local control of BM may not necessarily lead to improved survival but is of paramount importance to maintain neurological function and may be a worthwhile objective, especially in subsets of patients with a better prognosis. Treatment failure has been shown to have an impact on patient's neurocognitive function [4] and possibly quality of life (QoL) [5]. As such, selected subgroups of patients (i.e. younger age, good performance status, controlled primary tumor, absence of extracranial disease and/or limited number of BM) might benefit from dose escalation [2,6] although this strategy is historically controversial [7,8]. Any dose escalation paradigm may translate however in increased radiation-induced toxicity and may have a deleterious effect on neurocognitive function and/or QoL. Two studies assessed the QoL in patients with primary brain tumors and BMs [9,10], but no study has studied prospectively this end-point specifically for BM's patients treated with a dose-escalation paradigm. Consequently, we embarked in a prospective study assessing the toxicity, neurocognitive function and QoL of good prognostic patients with 1 - 4 BM treated with a dose escalation strategy using a volumetric modulated arc therapy technique (VMAT).

## Patients and methods

### Patients and Treatment characteristics

From March 2010 to December 2010, 29 patients with previously untreated brain metastasis were included into an institutional prospective protocol of WBRT with SIB to single or multiple BMs. Patient eligibility for this trial was as follows: histologically proven cancer; brain MRI consistent with BM(s); 1 to 4 BMs; age < 80 years; Karnofsky performance status (KPS) ≥ 70; RPA < III; total volume of BM ≤ 40 ml and no previous cranial RT. The presence of extracranial disease was allowed. Patients were allowed to undergo craniotomy. Quality-of-life (QoL) was evaluated prospectively using the QLQ-C30 and -BN20 instruments developed by the European Organization for Research and Treatment of Cancer (EORTC). The primary end-point of this study was QoL. The secondary endpoints were toxicity, overall survival (OS) and brain progression-free survival (PFS). The characteristics of the patients are detailed in Table 1.

**Table 1:** Patient's characteristics (*n *= 29)

	n	%
Gender		
Male	16	55.2
Female	13	44.8
		
Age (Years)		
Median	62.3 (range, 42-78.3)	
Mean	62.1 ± 8.5	
		
RPA		
I	6	20.7
II	23	79.3
		
GPA		
0 - 1	3	10.3
1.5 - 2.5	21	72.5
3	3	10.3
3.5 - 4	2	6.9
		
Primary Tumor		
Lung	22	76.0
Breast	3	10.3
Melanoma	1	3.4
Other	3	10.3
		
Number of BM		
1	13	44.8
2 - 3	9	30.1
4	7	24.1
		
Surgery	15	51.7
		
Concomitant chemotherapy	10	34.5

*Abbreviations *: RPA, Recursive Partitioning Analysis; GPA, Graded Prognosis Assesment; BM, Brain Metastasis.

The gross tumor volume (GTV) was defined as the BM and/or the surgical resection cavity. The planning target volume (PTV) was obtained by adding a 3 mm margin to the GTV. In association with WBRT, a SIB was administered to all brain lesions. A composite VMAT plan was generated for all patients, consisting of WBRT (30 Gy in 10 fractions) with a SIB of 10 Gy in 10 fractions to the PTVs. The cumulative dose delivered to the BM(s) was thus 40 Gy in 10 fractions. The treatment plans were generated using a volumetric modulated arc therapy (VMAT) technique, all computed on the Varian Eclipse treatment planning system with 6 MV photon beams from a Varian Clinac equipped with a Millennium Multileaf Collimator (MLC; Novalis Tx, BrainLab, Feldkirchen, Germany) with 120 leaves. Plans were optimized selecting a maximum dose rate of 600 MU/min. Two modulated arcs were used for all patients. Mean UM delivered was 671 ± 142.

Patients were treated using a thermoplastic immobilization mask used during simulation, with positioning determined by co-registration of the simulation kV CT scan with a MVCT scan acquired on the treatment unit.

### Quality-of-life questionnaires and administration

QoL in this study was assessed with the EORTC QLQ-C30 (version 3.0) and-QLQ-BN20. The self-administered QLQ-C30 is the EORTC core QoL questionnaire that addresses a range of functional outcomes and symptoms relevant to a wide range of cancer populations [11]. This 30-item questionnaire is composed of both multi-item scales and single-item measures. It is composed of a global health status (GHS) scale (2 items), functional scales (15 items) and symptom scales/items (13 items). Functional scales consist of physical (PF), role (RF), emotional (EF), cognitive and social functioning scales. Each item is scored from 1 to 4 (''not at all'': 1; ''a little'': 2; ''quite a bit'': 3; ''very much'': 4). As an exception, GHS is scored from 1 (''very poor'') to 7 (''excellent''). Raw scores (RS) were obtained by calculating the average of all item components. Item range is the difference between the maximum and minimum response to an individual item. The core calculation is detailed in the Appendix. A higher score for the GHS and functional scales represent thus a higher QoL and high level of functioning, respectively. Conversely, a high score for a symptom scale represents a high level of symptomatology.

The self-administered QoL questionnaire BN20 consists of 4 multi-items scale that assesses: future uncertainty (4 items), visual disorder (3 items), motor dysfunction (3 items) and communication deficit (3 items) [12]. Additionally, symptoms are addressed by single items: headaches, hair loss, weakness of legs and bladder control. The scoring algorithm for the scales is similar to the scoring of the EORTC-C30 questionnaire. RS are computed and linearly transformed to a 0 - 100 scale. For the scales and items, a higher score represents *worse *QoL. Details of the in-field testing of the BN20 in a multi-national and multi-lingual setting have been published previously [13].

The QoL assessment took place during the first consultation in the radiation oncology department, during VMAT (week 1 & 2) and every 3 months after the end of VMAT until tumor progression. All QLQ-C30 and -BN20 scores were prospectively collected into an institutional electronic database.

### Performance status and neurocognitive function

Performance status was assessed using the standard KPS scale [14]. Neurocognitive function was assessed using the MMSE dementia-scale [15], which has been shown to be a survival prognosticator in BM patients [16]. The baseline and follow-up evaluation of the KPS and MMSE was performed by the same attending radiation oncologist before the start of treatment, during and after VMAT.

### Radiation-induce toxicity

Alopecia was were classified according to the National Cancer Institute Common Terminology Criteria for Adverse Events (CTCAE) v3.0 grading system http://ctep.cancer.gov/search/search.asp?zoom_query = CTCAE&Action = Go%3E, except for skin toxicity which was scored using the Radiation Therapy Oncology Group (RTOG) scoring system http://www.rtog.org/ResearchAssociates/AdverseEventReporting/CooperativeGroupCommonToxicityCriteria.aspx. Toxicity assessment was made during VMAT (week 1 & 2) and every 3 months after the end of VMAT.

### Statistical analysis

OS and PFS were calculated using the Kaplan Meier method [17]. Recorded events were death (all causes of death included) and local and distant brain failure for OS and PFS, respectively. Survival differences between subgroups were evaluated using the log-rank test (*p *value < 0.05 was considered statistically significant). QoL results are presented as mean scores with standard deviations and were compared between time points using the Wilcoxon rank sum test and a *p *value < 0.05 was considered statistically significant. All analyses were performed using the SPSS statistical package (SPSS 17.0, Chicago, IL).

## Results

### Patients' outcome and prognostic factors

After a mean FU of 5.4 ± 2.8 months, 14 (48.3%) patients died. The 6-month OS was 55.1%. Patient undergoing surgery survived significantly longer than those not undergoing surgery: the estimated 6-month OS was 72.0% *vs*. 33.5%, respectively (*p *= 0.035). Likewise, patients with good performance status lived significantly longer. The estimated 6-month OS was 66.9% and 37.5% for patients with a KPS of 90-100 and 70-80, respectively (*p *= 0.025). Motor dysfunction (*p *= 0.11), emotional functioning (EF; *p *= 0.25), role functioning (RF; *p *= 0.27), future uncertainty (*p *= 0.35), global health status (GHS; *p *= 0.38), MMSE (*p *= 0.40), visual deficit (*p *= 0.59), number of BM (*p *= 0.64), age (*p *= 0.66), communication deficit (*p *= 0.79), gender (*p *= 0.80) and physical functioning (PF; *p *= 0.85) were however not prognostic for OS in this study.

Overall, 6 treatment failures were observed. Three (13.0%) patients presented with local failure but distant brain control and another 3 (13.0%) presented with local control but distant brain failure. The estimated 6-months brain PFS was 77.9%. Overall, 23 (79.4%) patients were controlled locally and distantly in the brain. The majority (*n *= 17 out of 23 tumour progression; 74.0%) presented with progressive extra-cranial systemic disease. Toxicity was minimal. No radiation-induced erythema was observed. Grade CTCAE 1 and 2 alopecia was only observed in 9 (31.0%) patients.

### QoL and neurocognitive function

**Figure 1:**
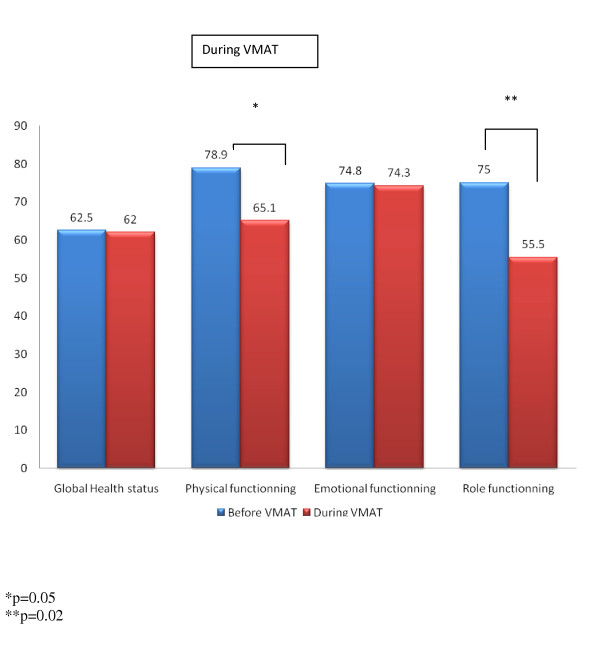
**Self-assessed QoL (EORTC-C30) before and during VMAT for Global Health Status, and physical, emotional and role functioning**.

MMSE, KPS and self-assessed QoL were compared during VMAT. Nineteen (65.5%) patients completed all questionnaires before and during VMAT (week 1 and 2). The reasons for not completing the questionnaires in the other 10 (34.5%) patients were as follow: accidental destruction of the QoL questionnaires by the administrative team in 5 patients, deterioration of cognitive function or performance status preventing completing of the questionnaires in 2 patients, non compliance in questionnaire administration by physicians in 2 patients and patient refusal in 1 patients. During VMAT, the performance status decreased although not significantly so. Mean KPS before and during VMAT was 89.4 ± 11.6 and 85.3 ± 18.1, respectively (*p *> 0.1). MMSE significantly improved however during VMAT. Mean MMSE scores were 27.1 ± 2.7 and 28.1 ± 2.5 (*p *= 0.04). During VMAT, GHS remained stable (Figure 1; Table 2). For the C30 functional scale, a statistical trend was observed for decreasing PF during VMAT (Figure 1; Table 2). EF remained fairly stable during VMAT (Figure 1; Table 2). RF was however significantly decreased during VMAT (Figure 1; Table 2). Table 2 details the domain's and single item's scores of the BN20 questionnaire during VMAT. Headaches were significantly decreased during VMAT. Mean headache-BN20 observed scores were 37.0 ± 41.0 and 18.5 ± 23.5 before and during VMAT, respectively (*p *= 0.048; Table 2). A statistical trend was observed for future uncertainty, which decreased during VMAT (Table 2; *p *= 0.07). Communication deficit also decreased during VMAT, although no statistical trend was observed (Table 2; *p *= 0.13). Interestingly, hair loss issues was reported less often during VMAT (Table 2; *p *= 0.11).

**Table 2:** EORTC-C30 and -BN20 domain and single item's scores before and during VMAT

QoL	Pre-VMAT score ± SD	Score during VMAT ± SD	*p *value
C30			
Global Health Status	62.5 ± 26.4	62.0 ± 21.4	0.92
Physical functioning	78.9 ± 22.9	65.1 ± 31.2	0.05
Emotional functioning	74.8 ± 20.1	74.3 ± 27.0	0.86
Role functioning	75.0 ± 21.6	55.5 ± 37.1	0.02
			
BN20 domains/items			
*Domains*			
Future uncertainty	30.8 ± 20.9	18.8 ± 23.5	0.07
Visual disorder	13.6 ± 20.7	13.3 ± 17.4	0.92
Motor dysfunction	22.5 ± 24.1	22.2 ± 26.9	0.64
Communication deficit	19.7 ± 28.1	13.6 ± 16.8	0.13
			
*Single items*			
Headache	37.0 ± 41.0	18.5 ± 23.5	< 0.05
Hair loss	14.8 ± 30.7	3.7 ± 15.7	0.11
Weakness of legs	24.1 ± 29.8	25.9 ± 31.4	0.58
Bladder control	16.7 ± 30.8	14.8 ± 28.5	0.79

*Abbreviations: *QoL: Quality of life; SD, standard deviation.

**Figure 2:**
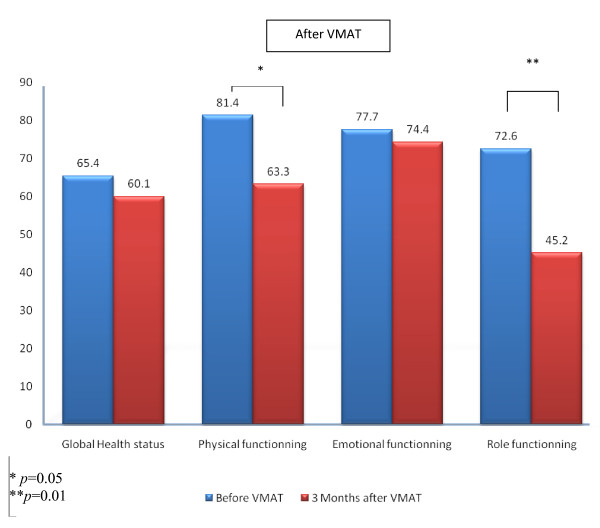
**Self-assessed QoL (EORTC-C30) before and 3 months after VMAT for Global Health Status, and physical, emotional and role functioning**.

MMSE, KPS and self-assessed QoL were also compared for the time points before and 3 months after the start of VMAT. Fourteen (77.8%) patients completed questionnaires at both time points. The reason for not completing the questionnaires in the other 4 (22.2%) patients was death within 3 months after VMAT in all patients. Among the patients completing the questionnaire, 4 (28.6%) presented with systemic progressive disease. Three brain failures (21.4%) were observed. At 3-months follow-up, patients had a significantly worse performance status. Mean KPS scores were 92.1 ± 8.0 and 82.1 ± 15.8 before and after VMAT, respectively (*p *= 0.01). MMSE score were however stable. Mean MMSE scores were 27.3 ± 2.4 and 27.4 ± 4.2 before and 3 months after VMAT, respectively (*p *= 0.33). GHS remained fairly stable (Figure 2; Table 3). For PF, a statistical trend was observed for decreasing values after VMAT (Figure 2; Table 3). EF remained also stable after VMAT (Figure 2; Table 3). RF was however significantly decreased (Figure 2; Table 3).

**Table 3:** EORTC-C30 and -BN20 domain and single item's scores before and 3 months after VMAT

QoL	Pre-VMAT scoren ± SD	Score during VMAT ± SD	*p *value
C30			
Global Health Status	65.4 ± 22.8	60.1 ± 26.6	0.72
Physical functioning	81.4 ± 17.6	63.3 ± 29.8	0.05
Emotional functioning	77.7 ± 21.2	74.4 ± 26.2	0.91
Role functioning	72.6 ± 22.3	45.2 ± 37.8	0.01
			
BN20 domains/items			
*Domains*			
Future uncertainty	27.1 ± 20.7	27.4 ± 31.6	0.66
Visual disorder	12.7 ± 21.3	15.1 ± 21.2	0.25
Motor dysfunction	21.4 ± 24.8	22.2 ± 33.5	0.96
Communication deficit	23.0 ± 30.3	27.8 ± 29.5	0.55
			
*Single items*			
Headache	33.3 ± 37.0	7.1 ± 14.2	< 0.05
Hair loss	14.3 ± 31.2	23.8 ± 30.5	0.48
Weakness of legs	21.4 ± 24.8	28.6 ± 34.2	0.39
Bladder control	21.4 ± 33.6	11.9 ± 24.8	0.26

*Abbreviations: *QoL: Quality of life; SD, standard deviation.

Table 3 details the domain's and single item's scores of the BN20 questionnaire. Except for headache and bladder control scores, all other scores worsened 3 months after VMAT. As mentioned, headaches were significantly decreased after VMAT. Mean headache-BN20 observed scores were 33.3 ± 37.0 and 7.1 ± 14.2 before and 3 months after VMAT, respectively (*p *= 0.046; Table 3). Interestingly, hair loss was reported more often 3 months after VMAT, although not significantly so. Mean hair loss-BN20 observed scores were 14.3 ± 31.2 and 23.8 ± 30.5 before and 3 months after VMAT, respectively (*p *= 0.48; Table 3).

MMSE, KPS and self-assessed QoL were also compared for the time points before and 6 months after the start of VMAT. Five (17.2%) patients completed questionnaires at both time points. All other patients with complete C30 and BN20 data did not reach this time point. In these patients, no local or distant brain failure was observed. One patient presented with progressive extra-cranial systemic disease. Mean KPS scores were 94.0 ± 5.5 and 90 ± 14.1 before and 6 months after VMAT, respectively (*p *= 0.41). Mean MMSE scores were 27.0 ± 2.4 and 27.8 ± 2.9 before and 6 months after VMAT, respectively (*p *= 0.18). EORTC-C30 scores remained stable (Table 4). Table 4 also details the domain's and single item's scores of the BN20 questionnaire. All but communication deficit domains were non-significantly worse at 6 months (Table 4).

**Table 4:** EORTC-C30 and -BN20 domain and single item's scores before and 6 months after VMAT

QoL	Pre-VMAT score ± SD	Score during VMAT ± SD	*p *value
C30			
Global Health Status	62.5 ± 26.4	68.8 ± 20.8	0.47
Physical functioning	78.9 ± 22.9.6	71.9 ± 26.0	0.72
Emotional functioning	74.8 ± 20.1	70.0 ± 41.5	0.89
Role functioning	75.0 ± 21.6	73.3 ± 41.8	0.72
			
BN20 domains/items			
*Domains*			
Future uncertainty	26.0 ± 23.5	30.0 ± 41.5	0.72
Visual disorder	15.5 ± 12.7	28.9 ± 36.5	0.41
Motor dysfunction	15.5 ± 16.8	24.4 ± 30.8	0.59
Communication deficit	22.2 ± 28.3	6.6 ± 6.1	0.29
			
*Single items*			
Headache	26.7 ± 43.5	20.0 ± 29.8	0.99
Hair loss	13.3 ± 29.8	0.0 ± 0.0	0.32
Weakness of legs	20.0 ± 18.2	26.6 ± 27.9	0.66
Bladder control	0.0 ± 0.0	0.0 ± 0.0	1.00

*Abbreviations: *QoL: Quality of life; SD, standard deviation.

## Discussion

To the best of our knowledge, the present study is the first series ever published on a prospective evaluation of QoL in patients with BM, not including primary brain tumors, treated with VMAT and dose escalation. The efforts at developing new therapeutic strategies in BM should not only focus on increasing survivorship but should also assess their relative impact on QoL. Dose escalation may be potentially neurotoxic and thus negatively affect health-related QoL in these BM patients. As such, we embarked in a careful prospective evaluation of QoL in a pilot VMAT dose escalation protocol. Our data suggests several comments. First, QoL assessment was indeed difficult in this patient cohort, even though a dedicated physician was assigned to do the QoL assessment. The difficulty in assessing QoL or neurocognitive function in patients with BM has been reported by other investigators [5,14]. Only one patient out of two could be assessed at the 3 months post-VMAT time point, as a result of decreasing performance status and/or cognitive function, administrative issues or patient refusal. Second, the overall QoL of these patients did indeed decrease 3 months after VMAT as reported by other authors [18](Table 3). In order to avoid any potential bias originating from the death of patients with poor-QoL, the comparison of QoL at the two time points (i.e. baseline and at 3 months) was performed using only data from patients completing questionnaires at both time points [19]. Additionally, performance status did also significantly decrease, but the neurocognitive function, assessed with the MMSE, appeared to be stable in this small cohort. Interestingly, the levels of three EORTC-BN20 domains, namely visual disorder, motor dysfunction and communication deficit, remained stable at this time point, suggesting a possible association between MMSE and these scores, as reported by other authors [13]. Third, except for physical and role functioning (Figure 1), the observed QoL did not substantially decrease during treatment. MMSE significantly increased during treatment, possibly as a result of the therapeutic effect of radiation. Although one third of patients experienced alopecia, the EORTC-BN20 mean score for this item was decreased (Table 2), suggesting that there is not necessarily agreement between patient and physician reports of symptoms or toxicity [20,21]. Some EORTC-BN20 domains were even improved during VMAT (Table 2), with an observed statistical trend for future uncertainty levels, which may reflect the effectiveness of coping strategies. Finally, lower functional and GHS scores in the EORTC-C30 questionnaire and higher EORTC-BN20 domain's scores were associated with decreased survival suggesting that these scores may be relevant prognosticators for BMs [18]. Although no statistical trend was observed, possibly as a result of small numbers, all Kaplan-Meier curves were parallel to what was expected, i.e. worse QoL was related to decreased survivorship (data not shown). Using traditional method of statistical analysis (i.e. Cox multivariate model) controlled for major clinical prognostic factors, some EORTC-C30 or -BN20 items, such as cognitive function or GHS, have been significantly associated with survival in two prospective trials [22,23], although these results are controversial [9,24,25]. The mechanisms underlying these potential associations are however unclear. QoL scores may reflect the patient's physical and psychological state that may have a positive effect on the overall disease process (i.e. higher QoL score are a proxy for the patient's health status that may have a positive effect on the underlying disease). Alternatively to this true causative relationship, these scores may reveal the early perception and severity of the disease more accurately than conventional prognostic indices (i.e. lower QoL score reflect a worse underlying disease). Of note, several issues, such as the intercorrelation of the QoL parameters or the high variability in survival in patients with identical QoL scores to name a few, have been raised by the use of classical methods of statistical computation [23]. Further research regarding the prognostic value of health-related QoL is justified in the framework of future prospective trials.

We sought to investigate whether a hypofractionated SIB approach for the treatment of patients with 1 - 4 BMs would be a safe alternative to WBRT with or without radiosurgery. The observed toxicity was minimal. Less than one third of patients had alopecia at the end of treatment. These results may be in keeping with recent phase I dose escalation studies reporting the toxicity in patients treated with WBRT and an SIB technique [26,27]. In the US study, alopecia and skin reaction were reported in 16% and 6% of patients, respectively [26]. The reduction of the observed alopecia rates, when compared to those observed with WBRT, may have an impact on the patient's QoL, as assessed by the EORTC-BN20 questionnaire (Table 4).

The treatment of patients with BM can consist of best supportive care, surgery or radiosurgery with or without WBRT. We have included patients with one BM in our treatment protocol as the modulation of the WBRT by a VMAT approach may produce steeper radiation-dose gradients than plans with conventional WBRT summed with radiosurgery dose deposition [28]. For patients with good- to intermediate-prognosis (i.e. RPA I - II), such as those treated in our protocol, a multi-modality treatment strategy is usually proposed with the aim of preventing intracranial progression, preserving the neurologic function and possibly the overall QoL. In our study, local and distant brain tumor control was achieved in a majority of patients. Unfortunately, systemic extracranial progression was observed in a majority (74.0%) of patients with a consequential impact on survivorship. Interestingly, the estimated 6-months OS was significantly increased (72% *vs*. 33.5%) when surgery was performed to patients with 1 - 4 BM. These results should be interpreted cautiously, as they may be subject to uncontrolled patient selection into different treatment groups (i.e. better KPS and RPA/GPA scores for patients undergoing surgery). They are however in line with several prospective studies confirming the importance of surgery in selected patients [29,30]. Although we did not perform a multivariate analysis as the number of events relative to the potential parameters was inappropriate, these data suggest that dose escalation only with a SIB technique may not be the optimal treatment for these good- to intermediate-prognosis patients.

There were several limitations of our study. First, the small sample size of 29 patients limited the statistical power to assess fully the QoL of BM patients treated with VMAT and to detect associations between survival and the EORTC-C30 and -BN20 parameters. Second, the rate of completion of the questionnaires in these severely ill patients, although identical to the compliance rate reported in the literature, was suboptimal. High compliance in questionnaire completion is difficult to achieve in severely ill patients as their condition deteriorates over time. Third, as the number of brain progressions was low, the impact of this event on patient's QoL at the 3 months time point was not assessable. This being said, this study was a prospective study with specific QoL endpoints and the BM patient cohort studied was homogeneous and represented a good- to intermediate-prognosis population for whom the QoL is of paramount importance.

In summary, the delivery of 40 Gy in 10 fractions using a VMAT technique was achieved with no significant toxicity. The majority of patients presented with extracranial progressive disease. Surgery and performance status were significant prognostic factors for survival. Although the QoL did not decrease significantly during treatment, a decrease of several EORTC-C30 and -BN20 parameters was observed at 3 months after VMAT.

## Abbreviations

GPA: Graded Prognostic Assessment; BM: brain metastasis; KPS: Karnofsky performance status; RPA: recursive partitioning analysis; RTOG: Radiation Therapy Oncology Group; CTCAE: National Cancer Institute Common Terminology Criteria for Adverse Events; VMAT: volumetric modulated arc therapy; QoL: Quality of Life; WBRT: Whole brain radiotherapy; EORTC: European Organization for research and Treatment of Cancer; MMSE: Mini Mental State Examination; GHS: Global Health Status; PF: Physical functioning; EF: Emotional functioning; RF: Role functioning.

## Competing interests

The authors declare that they have no competing interests.

## Authors' contributions

DCW was responsible for the primary concept and the design of the study; FC, ML, KM and DCW, performed the data capture and analysis. FC and DCW drafted the manuscript; DCW performed the statistical analysis; FC and DCW reviewed patient data; all authors revised the manuscript. All authors have read and approved the final manuscript.

## Supplementary Material

Additional file 1 **Appendix**. Equations for functional scale, GHS and symptom scales/items scores. Click here for file

## References

[B1] Nussbaum ES Djalilian HR Cho KH Hall WA Brain metastases. Histology, multiplicity, surgery, and survival Cancer 1996 78 8 1781 8 10.1002/(SICI)1097-0142(19961015)78:8<1781::AID-CNCR19>3.0.CO;2-U 8859192

[B2] Andrews DW Scott CB Sperduto PW Flanders AE Gaspar LE Schell MC Whole brain radiation therapy with or without stereotactic radiosurgery boost for patients with one to three brain metastases: phase III results of the RTOG 9508 randomised trial Lancet 2004 363 9422 1665 72 10.1016/S0140-6736(04)16250-8 15158627

[B3] Kondziolka D Patel A Lunsford LD Kassam A Flickinger JC Stereotactic radiosurgery plus whole brain radiotherapy versus radiotherapy alone for patients with multiple brain metastases International journal of radiation oncology, biology, physics 1999 45 2 427 34 10.1016/S0360-3016(99)00198-4 10487566

[B4] Meyers CA Smith JA Bezjak A Mehta MP Liebmann J Illidge T Neurocognitive function and progression in patients with brain metastases treated with whole-brain radiation and motexafin gadolinium: results of a randomized phase III trial J Clin Oncol 2004 22 1 157 65 14701778 10.1200/JCO.2004.05.128

[B5] Li J Bentzen SM Li J Renschler M Mehta MP Relationship between neurocognitive function and quality of life after whole-brain radiotherapy in patients with brain metastasis International journal of radiation oncology, biology, physics 2008 71 1 64 70 10.1016/j.ijrobp.2007.09.059 18406884

[B6] Casanova N Mazouni Z Bieri S Combescure C Pica A Weber DC Whole brain radiotherapy with a conformational external beam radiation boost for lung cancer patients with 1-3 brain metastasis: a multi institutional study Radiat Oncol 2010 5 13 10.1186/1748-717X-5-13 20167107 PMC2834695

[B7] Harwood AR Simson WJ Radiation therapy of cerebral metastases: a randomized prospective clinical trial International journal of radiation oncology, biology, physics 1977 2 11-12 1091 4 10.1016/0360-3016(77)90114-6 74375

[B8] Borgelt B Gelber R Kramer S Brady LW Chang CH Davis LW The palliation of brain metastases: final results of the first two studies by the Radiation Therapy Oncology Group International journal of radiation oncology, biology, physics 6 1 1 9 10.1016/0360-3016(80)90195-9 6154024

[B9] Sehlen S Lenk M Hollenhorst H Schymura B Aydemir U Herschbach P Quality of life (QoL) as predictive mediator variable for survival in patients with intracerebral neoplasma during radiotherapy Onkologie 2003 26 1 38 43 10.1159/000069862 12624516

[B10] Regine WF Schmitt FA Scott CB Dearth C Patchell RA Nichols RC Jr Feasibility of neurocognitive outcome evaluations in patients with brain metastases in a multi-institutional cooperative group setting: results of Radiation Therapy Oncology Group trial BR-0018 International journal of radiation oncology, biology, physics 2004 58 5 1346 52 10.1016/j.ijrobp.2003.09.023 15050309

[B11] Aaronson NK Ahmedzai S Bergman B Bullinger M Cull A Duez NJ The European Organization for Research and Treatment of Cancer QLQ-C30: a quality-of-life instrument for use in international clinical trials in oncology J Natl Cancer Inst 1993 85 5 365 76 10.1093/jnci/85.5.365 8433390

[B12] Osoba D Aaronson NK Muller M Sneeuw K Hsu MA Yung WK The development and psychometric validation of a brain cancer quality-of-life questionnaire for use in combination with general cancer-specific questionnaires Qual Life Res 1996 5 1 139 50 10.1007/BF00435979 8901377

[B13] Taphoorn MJ Claassens L Aaronson NK Coens C Mauer M Osoba D An international validation study of the EORTC brain cancer module (EORTC QLQ-BN20) for assessing health-related quality of life and symptoms in brain cancer patients Eur J Cancer 2010 46 6 1033 40 10.1016/j.ejca.2010.01.012 20181476

[B14] Komosinska K Kepka L Niwinska A Pietrzak L Wierzchowski M Tyc-Szczepaniak D Prospective evaluation of the palliative effect of whole-brain radiotherapy in patients with brain metastases and poor performance status Acta oncologica (Stockholm, Sweden) 2010 49 3 382 8 10.3109/02841860903352942 20397770

[B15] Folstein MF Folstein SE McHugh PR "Mini-mental state". A practical method for grading the cognitive state of patients for the clinician J Psychiatr Res 1975 12 3 189 98 10.1016/0022-3956(75)90026-6 1202204

[B16] Murray KJ Scott C Zachariah B Michalski JM Demas W Vora NL Importance of the mini-mental status examination in the treatment of patients with brain metastases: a report from the Radiation Therapy Oncology Group protocol 91-04 International journal of radiation oncology, biology, physics 2000 48 1 59 64 10.1016/S0360-3016(00)00600-3 10924972

[B17] Kaplan E Meier P Nonparametric estimation for incomplete observations J Am Stat Assoc 53 458 481

[B18] Steinmann D Schafer C van Oorschot B Wypior HJ Bruns F Bolling T Effects of radiotherapy for brain metastases on quality of life (QoL). Prospective pilot study of the DEGRO QoL working party Strahlenther Onkol 2009 185 3 190 7 10.1007/s00066-009-1904-0 19330297

[B19] Vordermark D Avoiding bias in the prospective evaluation of patients with brain metastases J Clin Oncol 2007 25 25 4023 author reply 4024-5 10.1200/JCO.2007.12.4610 17761992

[B20] Fromme EK Eilers KM Mori M Hsieh YC Beer TM How accurate is clinician reporting of chemotherapy adverse effects? A comparison with patient-reported symptoms from the Quality-of-Life Questionnaire C30 J Clin Oncol 22 17 3485 90 10.1200/JCO.2004.03.025 15337796

[B21] Basch E Iasonos A McDonough T Barz A Culkin A Kris MG Patient versus clinician symptom reporting using the National Cancer Institute Common Terminology Criteria for Adverse Events: results of a questionnaire-based study Lancet Oncol 2006 7 11 903 9 10.1016/S1470-2045(06)70910-X 17081915

[B22] Mauer M Stupp R Taphoorn MJ Coens C Osoba D Marosi C The prognostic value of health-related quality-of-life data in predicting survival in glioblastoma cancer patients: results from an international randomised phase III EORTC Brain Tumour and Radiation Oncology Groups, and NCIC Clinical Trials Group study British journal of cancer 2007 97 3 302 7 10.1038/sj.bjc.6603876 17609661 PMC2360328

[B23] Mauer ME Taphoorn MJ Bottomley A Coens C Efficace F Sanson M Prognostic value of health-related quality-of-life data in predicting survival in patients with anaplastic oligodendrogliomas, from a phase III EORTC brain cancer group study J Clin Oncol 2007 25 36 5731 7 10.1200/JCO.2007.11.1476 18089867

[B24] Meyers CA Hess KR Yung WK Levin VA Cognitive function as a predictor of survival in patients with recurrent malignant glioma J Clin Oncol 18 3 646 50 10.1200/JCO.2000.18.3.646 10653880

[B25] Klein M Postma TJ Taphoorn MJ Aaronson NK Vandertop WP Muller M The prognostic value of cognitive functioning in the survival of patients with high-grade glioma Neurology 2003 61 12 1796 8 14694052 10.1212/01.wnl.0000098892.33018.4c

[B26] Rodrigues G Yartsev S Yaremko B Perera F Dar AR Hammond A Phase I Trial of Simultaneous In-Field Boost With Helical Tomotherapy for Patients With One to Three Brain Metastases International journal of radiation oncology, biology, physics 2010 10.1016/j.ijrobp.2010.03.047 20675078

[B27] Bauman G Yartsev S Fisher B Kron T Laperriere N Heydarian M Simultaneous infield boost with helical tomotherapy for patients with 1 to 3 brain metastases American journal of clinical oncology 2007 30 1 38 44 10.1097/01.coc.0000245473.41035.c4 17278893

[B28] Lagerwaard FJ van der Hoorn EA Verbakel WF Haasbeek CJ Slotman BJ Senan S Whole-brain radiotherapy with simultaneous integrated boost to multiple brain metastases using volumetric modulated arc therapy International journal of radiation oncology, biology, physics 2009 75 1 253 9 10.1016/j.ijrobp.2009.03.029 19577856

[B29] Patchell RA Tibbs PA Walsh JW Dempsey RJ Maruyama Y Kryscio RJ A randomized trial of surgery in the treatment of single metastases to the brain The New England journal of medicine 1990 322 8 494 500 10.1056/NEJM199002223220802 2405271

[B30] Noordijk EM Vecht CJ Haaxma-Reiche H Padberg GW Voormolen JH Hoekstra FH The choice of treatment of single brain metastasis should be based on extracranial tumor activity and age International journal of radiation oncology, biology, physics 1994 29 4 711 7 10.1016/0360-3016(94)90558-4 8040016

