# Supplementary material for: Simultaneous in-field boost for patients with 1 to 4 brain metastasis/es treated with volumetric modulated arc therapy: a prospective study on quality-of-life
Source: Radiat Oncol. 2011 Jun 30;6:79. doi: 10.1186/1748-717X-6-79 (PMC3158112; doi:10.1186/1748-717X-6-79)
Supplement: Additional file 1 — Appendix. Equations for functional scale, GHS and symptom scales/items scores. [file 1748-717X-6-79-S1.DOC]

**APPENDIX**

For functional scales, the score is obtained using the formula:
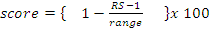
. For GHS and symptom scales / items, the following formula is employed:
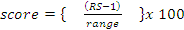
.
